# Early prognostic factors in distal radius fractures in a younger than osteoporotic age group: a multivariate analysis of trauma radiographs

**DOI:** 10.1186/1471-2474-14-170

**Published:** 2013-05-22

**Authors:** Annechien Beumer, Tommy R Lindau, Catharina Adlercreutz

**Affiliations:** 1 Department of Orthopaedics, Amphia Hospital, Breda, The Netherlands; 2 2Pulvertaft Hand Centre, Derby, UK; 3 Department of Radiology Lund, University Hospital, Lund, Sweden

**Keywords:** Ddistal radius fracture, Prognostic factor, Radiology, Outcome

## Abstract

**Background:**

Treatment of distal radius fractures in patients of a younger than osteoporotic age is complex, because they often are the result of a high-energy trauma and have intra-articular fractures and associated injuries. As yet no fracture classification exists that predicts outcome. Our aim was to find the earliest possible prognostic factor by testing which radiological parameter on the trauma radiograph would have the greatest impact on clinical outcome in a younger than osteoporotic age group.

**Methods:**

We assessed 66 patients (34 F) with unilateral fractures of the distal radius from a non-Osteoporotic age group. The median age was 42 years, (10^th^ -90^th^ percentile 20–54). Pre-reduction antero-posterior and lateral wrist radiographs were obtained and fracture pattern, radiocarpal joint surface tilt, radial length, radial inclination and ulnar variance were measured. Clinical outcome was assessed with the subjective part as well as the complete modified Gartland and Werley score. Multivariate analysis of those parameters was performed to assess which radiological parameter would best predict outcome.

**Results:**

It was found that post-traumatic ulna + (>2 mm) was the single factor that significantly correlated with a bad outcome. An intra-articular fracture pattern may also be a strong marker; however this was not statistically significant (RR 95% conf interval 0.94 – 20.59).

**Conclusions:**

The present study showed that post-traumatic ulna + is the most important factor in predicting bad outcome in non-osteoporotic patients, but that especially intra-articular fractures and to a lesser extent dorsal tilt may be of importance too.

## Background

It is known that distal radius fractures in patients of the non-osteoporotic age usually result from high energy trauma and often have intra-articular involvement [1,2]. Furthermore, they are associated with a higher incidence of cartilage, inter-carpal ligament and TFCC lesions [3,4]. These fractures are correlated with a worse outcome when instability of the distal radioulnar joint is present [5]. This instability does not correlate with radiographic features at the time of the trauma or at follow-up but to the presence of arthroscopically diagnosed peripheral TFCC-tears [6,7].

In order to predict, and thereby hopefully prevent, a bad outcome in non-osteoporotic fractures it is important to find the earliest prognostic markers for general bad outcome. These are patient characteristics and radiographic measurements on the traumaradiograph. The aim of this study was to assess the most objective parameters on the initial radiograph; these are i.e. radiocarpal joint surface tilt, radial length, radial inclination, ulnar variance. Furthermore we assessed more subjective radiologic parameters such as comminution as described in the subclasses of the AO-classification [8] and fractures through the ulnar styloid or the distal radio-ulnar or radiocarpal joint surfaces. In order to correlate these findings with functional outcome these findings were correlated with objective and subjective clinical outcome as assessed with the modified Gartland and Werley score [9,10] at 2 years (middle long) follow-up of distal radius fractures in a younger than osteoporotic age group. We correlated the radiological findings with both the subjective part as well as the complete modified Gartland and Werley score to assess whether the subjective part alone could be used as a patient reported outcome measure.

## Methods

**Figure 1:**
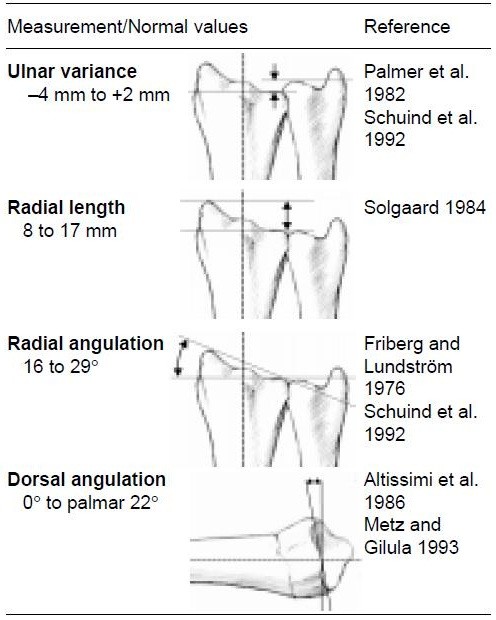
Radiographic Parameters.

During a consecutive year, all patients with distal radial fractures from a non-osteoporotic age group (men 20–59, women 20–49 years of age), without risk factors for osteoporosis, such as alcoholism, steroid use or early menopause, were seen by the same team of orthopaedic surgeons at the department of orthopaedics of Lund University Hospital, Sweden. Pre-reduction Antero-Posterior (AP) and lateral wrist radiographs were obtained and radiocarpal joint surface tilt, radial length, radial inclination and ulnar variance were measured (Figure 1). The normal value for radiocarpal joint surface tilt (dorsal angulation) is from 0° to palmer 22° [11-13], radial inclination (or angulation) ranges from 19° to 29° [12,14], radial length normally is between 8 and17 [13,15], and ulnar variance ranges from minus 4 to plus 2 mm [14,16]. Therefore palmar angulation exceeding 22° volar tilt or dorsal angulation tilted dorsally of the zero level, radial length less than 8 mm and radial inclination less than 19° or exceeding 29° as well as ulnar variance exceeding plus 2 mm (Ulna+) were considered pathological. Furthermore, assessment was done whether comminution [8], or fractures through the ulnar styloid or the distal radio-ulnar or radiocarpal joint surfaces were present. Those fracture criteria that could not be quantified in degrees of dislocation but merely as present/absent or more or less severe were considered ‘subjective radiologic criteria’. A logistic regression analysis of the above mentioned parameters on the initial trauma radiographs was performed to assess which parameters would have the greatest impact on clinical outcome as assessed with the subjective part of the modified Gartland and Werley score as well as the complete modified Gartland and Werley score ([9,10], Appendix 1). This study was conducted in accordance with the guidelines published by the Swedish Research Council and the International Committee of Medical Journal Editors and supported by the institutional review board of Lund University Hospital. Written informed consent was obtained from the patient for publication of this report and any accompanying images.

## Results

92 patients with 95 distal radial fractures were included. Thirteen patients were lost to follow-up, 10 patients did not have trauma radiographs because of immediate reduction in the emergency department making it impossible to include them in this study and 3 had to be excluded because of bilateral fractures and/or mental disability. This left 66 patients (34 F) with unilateral fractures of the distal radius. The median age was 42 years, (10^th^ -90^th^ percentile 20 –54). Fifty-seven fractures were treated with plaster, 25 of those had needed closed reduction. Nine fractures were treated surgically. Median time of immobilization was 4 weeks (10–90 percentile 3–6 weeks). The median follow-up was 27 (10–90 percentile 16–36) months. Post-traumatic ulna + (>2 mm) was the only statistically significant of all factors predicting outcome in both subjective (subjective part of Gartland and Werley Score) and objective-subjective (i.e. complete Gartland and Werley Score) clinical assessment according to the logistic regression analysis (Table 1). A fracture through the radiocarpal joint was a statistically significant factor when only subjective radiologic parameters (comminution, fractures through radiocarpal or radioulnar joint surface or fracture of the ulnar styloid) were correlated with objective-subjective assessment (complete Gartland Werley score), but not when the entire group of radiologic parameters was correlated with objective-subjective assessment (Tables 2 and 3).

**Table 1:** Objective and subjective radiologic parameters correlated with objective-subjective assessment (complete Gartland Werley score)

**Factor**	**Odds ratio^1^**	**95%**	**Conf Int p-value**
Ulna +	2.53	1.18 – 5.41	0.017
Radiocarpal #	4.41	0.94 – 20.59	0.059
Dorsal Tilt	1.06	0.99 – 1.14	0.073
Radial Length	0.97	0.43 – 2.17	0.937
Radial Inclination	1.13	0.73 – 1.73	0.588
Proc Styl Ulna #	2.02	0.37 – 11.06	0.416
DRUJ #	4.09	0.65 – 25.73	0.134
Comminution	2.57	0.30 – 21.50	0.384

^1^. Adjusted for differences in age and sex.

+ Positive ulnar variance.

# Fracture.

**Table 2:** Subjective radiologic parameters correlated with objective-subjective assessment (complete Gartland Werley score)

**Factor**	**Odds ratio^1^**	**95% Conf Int**	**p-value**
Radiocarpal #	2.57	0.84 – 7.89	0.099
Proc Styl Ulna #	2.27	0.71 – 7.19	0.165
DRUJ #	2.30	0.60 – 8.83	0.224
Comminution	0.44	0.11 – 1.72	0.236

^1^. Adjusted for differences in age and sex.

+ Positive ulnar variance.

# Fracture.

**Table 3:** Objective and subjective radiologic parameters correlated with subjective assessment (subjective part of Gartland Werley score)

**Factor**	**Odds ratio^1^**	**95%**	**Conf Int p-value**
Ulna +	1.05	1.01 – 3.26	0.045
Radiocarpal #	1.89	0.56 – 6.37	0.306
Dorsal Tilt	1.05	0.99 – 1.11	0.134
Radial Length	1.17	0.59 – 2.30	0.654
Radial Inclination	0.93	0.66 – 1.33	0.704
Proc Styl Ulna #	0.94	0.21 – 4.27	0.938
DRUJ #	1.73	0.36 – 8.33	0.493
Comminution	2.57	0.46 -20.29	0.250

^1^. Adjusted for differences in age and sex.

+ Positive ulnar variance.

# Fracture.

## Discussion

Our aim was to study the earliest possible prognostic radiologic factor regarding outcome for distal radius fractures in non-osteoporotic patients. Therefore we studied radiological parameters on the initial trauma-radiographs. Our patients were followed for a median follow-up of 27 months. We consider this a relevant follow-up for the purpose of this study as literature shows that improvement after distal radius fracture is mainly in the first year [17,18]. We studied the entire group of distal radius fractures and not just those treated in plaster or surgically because we searched for the earliest prognostic factor regardless of treatment. It was found that post-traumatic ulna + (>2 mm) was the only statistically significant factor that could predict a bad outcome in these non-osteoporotic patients in our setting. This was found both when the subjective part of modified Gartland and Werley score and the complete modified Gartland and Werley score were assessed. When the complete modified Gartland and Werley score was tested we also found dorsal tilt and intra-articular fractures through the radiocarpal joint surface to be factors of importance in the final outcome. These factors didn’t come out with statistically significant values, analyzing the confidence intervals, however, suggest them to be of importance (Table 1).

Other parameters that describe fracture dislocation (radial length and radial inclination) as well as comminution, fracture through the radiocarpal, radioulnar joint surfaces and ulnar styloid did not correlate with a bad clinical outcome after distal radius fractures in this non-osteoporotic age group. This is in agreement with the literature where the commonly used fracture classifications for distal radius fractures have not shown any correlation with clinical outcome [19-21]. This may be because intra-articular fractures are correlated with associated injuries, such as ligamentous injuries leading to intercarpal injuries [22,23] and TFCC tears causing late DRU-joint instability [2,6,7]. Consequently, classifications based on these measurable angles and fracture distributions are not of clinical use, which is further supported by the fact that inter,-and intra observer reliability is poor [24,25].

It is know that patients with intra-articular fractures with more than 2 mm incongruence have been found to get joint degeneration after 6.7 years [26], the same goes for fractures with more than 10° of dorsal tilt [27,28] and carpal malalignment [29,30]. We therefore advocate to rather use these prognostic factors than classifications in the normal clinical management of these fractures. In research studies, however, there may still be a need to consider using classifications. It could be postulated that a fracture classification involving carpal alignment and presence of ligament and TFCC injury might better predict outcome. This would be an interesting topic for further research.

The subjective part and the combined evaluation of the Gartland and Werley score were tested separately to assess if subjective evaluation alone of the patient would give as much information as the combined evaluation, which would allow for longer follow-up with questionnaires only in the future. In this respect we found the same significance regarding the post-traumatic ulna + (2 mm) to be the only relevant factor suggesting that in the future we may simplify outcome measurements to the subjective part of the Gartland and Werley score (Tables 1 and 3).

It can be argued that positive ulnar variance on a pre-reduction radiograph is not only the result of severe compression and dorsal tilting of the distal radius because of the fracture but instead of a pre-existing longer ulna before the injury. The only way to ascertain if positive ulnar variance was present before injury would have been to obtain radiographs of the contra lateral wrist and assess ulna length. We, as well as most emergency units in the world, choose not to do so because this is not, and will not become, common practice in the treatment of this frequent fracture. However, one might consider obtaining radiographs of the other wrist if there is still an ulna + after reduction to a well aligned radius without any signs of shortening, hereby checking whether there has been a positive ulnar variance before the radius fracture. The majority of our patients were treated non-surgically based on the clinical judgment of the surgeon on call, which is in accordance with common practice in the Scandinavian area in the second half of the past decade [31]. It might be postulated that more aggressive treatment, such as (fixed angle) volar plate fixation, might improve the end result in such a group of patients. This does not change the outcome that ulna + is the most important prognostic factor. It may, however, sharpen the indication for the use of the volar plates.

## Conclusion

The present study shows that intra-articular fractures and to a lesser extent dorsal tilt may be of importance, but that post-traumatic ulna + is the most important factor in predicting bad outcome after distal radius fracture in the non-osteoporotic age group (Table 1).

## Appendix 1 modified Gartland and Werley score

(Gartland and Werley 1952, Sarmiento et al. 1975)

Subjective evaluation (range 0 to 6 points)

Excellent -no pain, disability, or limitation of motion 0p

Good -occasional pain, slight limitation of motion, and no disability 2p

Fair -occasional pain, some limitation of motion, feeling of weakness in wrist, no particular disability if careful, and activities slightly restricted 4p

Poor -pain, limitation of motion, disability, and activities more or less markedly restricted 6p

Residual deformity (range 0 to 3 points)

Prominent ulnar styloid 1p

Residual dorsal tilt 2p

Radial deviation of hand 2-3p

Objective evaluation (range 0 to 5 points)

Loss of dorsiflexion (<45°) 5p

ulnar deviation (<15°) 3p

supination (<50°) 2p

pronation (<50°) 2p

palmarflexion (<30°) 1p

radial deviation (<15°) 1p

circumduction 1p

Pain in distal radio ulnar joint 1p

Grip strength −60% or less than the opposite side 1p

Complications (range 0–5 points)

Arthritic change

minimum 1p

minimum with pain 3p

moderate 2p

moderate with pain 4p

severe 3p

severe with pain 5p

Nerve complications (median) 1-3p

Poor fingerfunction due to cast 1-2p

FINAL RESULTS (ranges of points)

Excellent 0-2p

Good 3-8p

Fair 9-20p

Poor > 21p

## Competing interests

The authors declare that they have no competing interests.

## Authors’ contributions

TL designed the study, acquired data, analyzed results and revised the manuscript. CA interpreted radiographs, analyzed results and revised the manuscript. AB analyzed results and wrote and revised the manuscript. All authors read and approved the final manuscript.

## Pre-publication history

The pre-publication history for this paper can be accessed here:

http://www.biomedcentral.com/1471-2474/14/170/prepub
